# Carmna: classification and regression models for nitrogenase activity based on a pretrained large protein language model

**DOI:** 10.1093/bib/bbaf197

**Published:** 2025-04-24

**Authors:** Anqiang Ye, Ji-Yun Zhang, Qian Xu, Hai-Xia Guo, Zhen Liao, Hongtu Cui, Dongdong Zhang, Feng-Biao Guo

**Affiliations:** Department of Respiratory and Critical Care Medicine, Zhongnan Hospital of Wuhan University, School of Pharmaceutical Sciences, Wuhan University, 185 Donghu Road, Wuchang District, Wuhan 430071, China; Key Laboratory of Combinatorial Biosynthesis and Drug Discovery, Ministry of Education, Wuhan University, 185 Donghu Road, Wuchang District, Wuhan 430071, China; Department of Respiratory and Critical Care Medicine, Zhongnan Hospital of Wuhan University, School of Pharmaceutical Sciences, Wuhan University, 185 Donghu Road, Wuchang District, Wuhan 430071, China; Key Laboratory of Combinatorial Biosynthesis and Drug Discovery, Ministry of Education, Wuhan University, 185 Donghu Road, Wuchang District, Wuhan 430071, China; Department of Respiratory and Critical Care Medicine, Zhongnan Hospital of Wuhan University, School of Pharmaceutical Sciences, Wuhan University, 185 Donghu Road, Wuchang District, Wuhan 430071, China; Key Laboratory of Combinatorial Biosynthesis and Drug Discovery, Ministry of Education, Wuhan University, 185 Donghu Road, Wuchang District, Wuhan 430071, China; Department of Respiratory and Critical Care Medicine, Zhongnan Hospital of Wuhan University, School of Pharmaceutical Sciences, Wuhan University, 185 Donghu Road, Wuchang District, Wuhan 430071, China; Key Laboratory of Combinatorial Biosynthesis and Drug Discovery, Ministry of Education, Wuhan University, 185 Donghu Road, Wuchang District, Wuhan 430071, China; Department of Respiratory and Critical Care Medicine, Zhongnan Hospital of Wuhan University, School of Pharmaceutical Sciences, Wuhan University, 185 Donghu Road, Wuchang District, Wuhan 430071, China; Key Laboratory of Combinatorial Biosynthesis and Drug Discovery, Ministry of Education, Wuhan University, 185 Donghu Road, Wuchang District, Wuhan 430071, China; Department of Respiratory and Critical Care Medicine, Zhongnan Hospital of Wuhan University, School of Pharmaceutical Sciences, Wuhan University, 185 Donghu Road, Wuchang District, Wuhan 430071, China; Key Laboratory of Combinatorial Biosynthesis and Drug Discovery, Ministry of Education, Wuhan University, 185 Donghu Road, Wuchang District, Wuhan 430071, China; Department of Respiratory and Critical Care Medicine, Zhongnan Hospital of Wuhan University, School of Pharmaceutical Sciences, Wuhan University, 185 Donghu Road, Wuchang District, Wuhan 430071, China; Key Laboratory of Combinatorial Biosynthesis and Drug Discovery, Ministry of Education, Wuhan University, 185 Donghu Road, Wuchang District, Wuhan 430071, China; Department of Respiratory and Critical Care Medicine, Zhongnan Hospital of Wuhan University, School of Pharmaceutical Sciences, Wuhan University, 185 Donghu Road, Wuchang District, Wuhan 430071, China; Key Laboratory of Combinatorial Biosynthesis and Drug Discovery, Ministry of Education, Wuhan University, 185 Donghu Road, Wuchang District, Wuhan 430071, China

**Keywords:** nitrogenase activity, bio-fertilizer, pretrained large model, machine learning, interpretability analysis

## Abstract

Nitrogen-fixing microorganisms play a critical role in the global nitrogen cycle by converting atmospheric nitrogen into ammonia through the action of nitrogenase (EC 1.18.6.1). In this study, we employed six machine learning algorithms to model the classification and regression of nitrogenase activity (Carmna). Carmna utilized the pretrained large-scale model ProtT5 for feature extraction from nitrogenase sequences and incorporated additional features, such as gene expression and codon preference, for model training. The optimal classification model, based on XGBoost, achieved an average area under receiver operating characteristic curve of 0.9365 and an F1 score of 0.85 in five-fold cross-validation. For regression, the best-performing model was a stacking approach based on support vector regression, with an average R^2^ of 0.5572 and a mean absolute error of 0.3351. Further interpretability analysis of the optimal regression model revealed that not only the proportion and codon preferences of standard amino acids, but also the expression levels and spatial distance of nitrogenase genes were associated with nitrogenase activity. We also obtained the minimum nitrogen-fixing *nif* cluster. This study deepens our understanding of the complex mechanisms regulating nitrogenase activity and contributes to the development of efficient bio-fertilizers.

## Introduction

According to the *State of Food Security and Nutrition in the World* report, published by five United Nations specialized agencies, ~733 million people were projected to experience hunger in 2023. This equates to 1 in every 11 people globally and 1 in every 5 people in Africa [1]. Addressing food insecurity is an urgent issue. Furthermore, global warming has exacerbated the challenges to food production. The global use of nitrogen fertilizers has surged to meet the increasing demand for food. However, excessive and inefficient use of these fertilizers contributes to global warming and environmental eutrophication, leading to ecological disruptions [2]. Nitrogen-fixing microorganisms, which convert atmospheric nitrogen into ammonia through nitrogenase enzymes, play an important role in the nitrogen cycle. By utilizing nitrogen-fixing microorganisms as bio-fertilizers, it is possible to not only enhance crop yields but also mitigate environmental harm, promoting sustainable agricultural practices.

Nitrogenase is a metalloenzymatic complex, and there are three primary types: molybdenum, vanadium, and iron nitrogenases. Among these, the molybdenum-based nitrogenase is the most prevalent and widely studied. It is commonly found in a range of nitrogen-fixing microorganisms, including bacteria and cyanobacteria. In contrast, vanadium and iron nitrogenases are expressed in environments where molybdenum is scarce, such as in some anaerobic or sulfate-reducing bacteria [3]. However, the precise mechanism of action of nitrogenase remains complex and not fully understood. Nitrogenase activity is typically measured through acetylene reduction assays [4]. These enzymes are highly sensitive to oxygen, which inhibits their activity, necessitating complex protective mechanisms within the host microorganisms [5]. Despite significant progress in understanding the genetics and biochemistry of nitrogenase, the practical application of these insights in engineering robust nitrogen-fixing strains remains limited. Some scientists have searched for key functional pathways of biological nitrogen fixation by constructing metabolic network models [6–8]. We hope to use more advanced computational methods to explore the key factors that influence nitrogenase activity, as a guide to the design of efficient nitrogenase and more usable minimal nitrogen-fixing gene clusters.

Catalytic reaction equation for molybdenum nitrogen-fixing enzyme:


(1) \begin{equation*} {N}_2+8{H}^{+}+8{e}^{-}+16 ATP\to 2N{H}_3+{H}_2+16 ADP+16{P}_i. \end{equation*}

Machine learning (ML) provides new perspectives for elucidating the mechanisms of nitrogenase. It excels at addressing complex problems in high-dimensional spaces that are beyond human perception, enabling the discovery of underlying patterns in intricate data [9]. In the field of protein design, the application of artificial intelligence (AI) is rapidly expanding [10–12]. For example, Luo’s team employed unsupervised generative models to predict the adaptability of protein variants, guiding the design of enzyme variants with enhanced activity by leveraging the capabilities of ML models to explore high-dimensional sequence spaces [13]. Additionally, David Baker’s team developed RoseTTAFold, a deep learning-based structural prediction tool, which can navigate sequence space to generate novel proteins that both conform to specific structural requirements and meet sequence constraints [14].

Natural language processing (NLP) has made significant strides in automated text processing and linguistic analysis in recent years, and its applications have expanded to various disciplines, including bioinformatics. Recently, there has been a growing interest in using NLP-inspired unsupervised training for protein language models to extract features from large datasets of protein sequences [15]. Remarkably, these large-scale, pretrained protein models have been able to generate features that accurately capture the intrinsic structural and functional properties of proteins [16]. One notable example is ProtTrans [17], which leverages the UniRef and Big Fantastic Database (BFD) as corpora and combines two autoregressive models (e.g. Transformer-XL, GPT-2) with four autoencoder models [e.g. BERT, Text-To-Text Transfer Transformer (T5)] to produce protein representations through the self-attention mechanism of deep learning [18, 19].

Most amino acids are encoded by multiple codons. However, these synonymous codons are often used with varying frequencies, a phenomenon known as codon usage bias (CUB) [20]. This bias differs significantly across organisms and is influenced by both intrinsic and extrinsic factors. CUB not only impacts translation efficiency and gene expression levels but is also closely linked to the growth rates of organisms [5]. Studies have indicated that fast-growing organisms tend to favor optimized codons in highly expressed genes, promoting a more rapid and efficient translation process [21].

**Figure 1:**
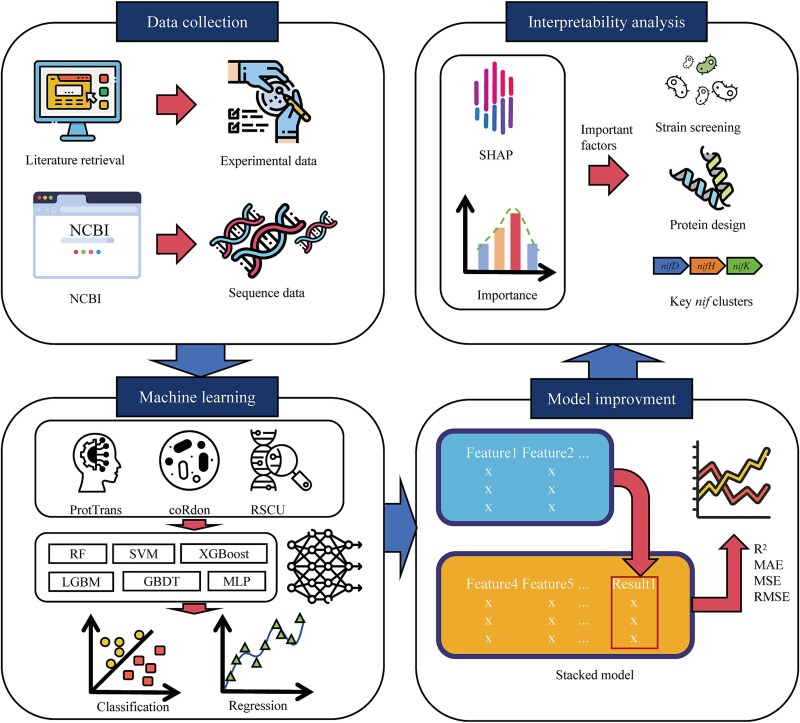
Overview of construction of Carmna.

In this study, we developed an Extreme Gradient Boosting (XGBoost)-based classification model and a support vector regression (SVR) stacking-based regression model [classification and regression models for nitrogenase activity (Carmna)] using features such as ProtTrans embeddings, gene expression, and codon preference to predict the nitrogenase activity efficiency of various strains (Fig. 1). Interpretive analysis of Carmna revealed five key standard amino acids that enhance nitrogenase activity; identified two key genes, *nifD* and *nifK*, which require a high expression for efficient nitrogen fixation; and explored how different codon preferences influence nitrogen fixation. The predictive models for nitrogenase activity can assist agricultural scientists and microbiologists in screening strains with high nitrogen-fixing capabilities, optimizing the genetic engineering design of nitrogen-fixing microorganisms and ultimately increasing crop yields while reducing the use of chemical nitrogen fertilizers. We also determined the minimal set of *nif* genes necessary for nitrogen fixation. These findings support the development of a screening method for strains with advantageous nitrogen fixation traits.

## Materials and methods

### Data collection and preprocessing

We collected nitrogenase activity values from the literature, measured using the acetylene reduction assay [22] and thoroughly searched through Google Scholar. To ensure consistency, all activity values were standardized to units of nmol C₂H₄/mg protein/hour and median values from multiple experimental results were used. Genomic data for nitrogen-fixing bacteria, along with corresponding nitrogenase protein sequences, were downloaded from the NCBI database. This resulted in a final dataset of 402 entries representing various strains of nitrogenase. To categorize enzyme activity, we set a threshold of 50 nmol C₂H₄/mg protein/hour: values above this threshold were labeled as positive (indicating high activity) and values below as negative (indicating low activity). Based on this classification, the dataset contained 209 negative samples and 193 positive samples. For model evaluation, we used stratified sampling, with 80% of the dataset allocated to the training set (167 negative and 154 positive samples) and the remaining 20% to the test set (42 negative and 39 positive samples). Model performance was assessed using a five-fold cross-validation method. To improve regression model training, we also adjusted nitrogenase activity values to minimize large labeling gaps that could hinder the model’s predictive accuracy:


(2) \begin{equation*} y={\log}_{10}\left(x+2\right), \end{equation*}

where *x* is the experimental data and *y* is the regression label.

In the following sections (Sequence-based coding scheme and Computation of codon preferences), we will describe how to extract features for each sample, which are used to train and test the classifying and regression models.

### Sequence-based coding scheme

ProtTrans is an advanced suite of protein language models designed to extract deep structural and functional insights from extensive protein sequence data. This suite includes two autoregressive and four autoencoding models based on a Transformer-based deep learning framework [23], which leverages self-attention mechanisms to capture intricate sequence features. ProtTrans is trained on a vast dataset containing up to 393 billion residues from the UniRef and BFD, significantly enhancing its generalization capacity and allowing it to excel across various protein function prediction tasks. In our study, we utilized the ProtT5 model (ProtT5-XL-UniRef50), obtained from the ProtTrans GitHub repository. ProtT5 is built upon the T5-XL architecture and is specifically trained on the UniRef50 database [24]. This model is designed to extract high-level features from protein sequences, producing an L × 1024-dimensional feature matrix for a sequence of length L. To streamline the use of these features in downstream applications, we averaged across the length dimension to generate a fixed 1 × 1024-dimensional vector, facilitating further analysis and model integration.

To infer the coding scheme of the triplet signature, we clustered the 20 amino acids into 7 classes based on side-chain dipole and volume properties [25]. Using this classification, we calculated the occurrence frequencies of any three consecutive groups of amino acids within each protein sequence [26]. This approach allows us to quantify the triplet signature composition of a sequence as a 343-dimensional feature vector (7 × 7 × 7), representing the varied chemical environments within protein structures.

Dipeptide composition (DPC) represents the frequency of occurrence of two consecutive amino acids, or dipeptides, throughout a protein sequence, converting the sequence into a 400-dimensional feature vector [27, 28]. DPC captures the composition of all adjacent amino acid pairs, with each possible dipeptide combination among the 20 natural amino acids forming the basis of the vector. This approach provides a 400-dimensional feature space (20 × 20), offering a comprehensive representation of local amino acid pair interactions within the protein sequence.

Pseudo-amino acid composition (PAAC) is widely utilized in proteomics to extract both physicochemical and compositional information from protein sequences, offering a more nuanced feature set for sequence characterization [29, 30]. In our work, we utilized PAAC to extract features, resulting in a 50-dimensional vector for each protein, comprising 20 dimensions for amino acid composition and 30 for pseudocomposition. This combination allows for a more robust and comprehensive analysis of protein sequences.

### Computation of codon preferences

We calculated the relative synonymous codon usage (RSCU) [31] and Euclidean distance (ECD), a commonly used distance metric that measures the straight-line distance between two points in multidimensional space [32]. RSCU values measure the frequency of different synonymous codon usage, reflecting an organism’s preference for certain codons in protein encoding. We calculated the ECD of the target species relative to the standard species without codon preference (all codon preference values are 1). ECD was calculated using the following formula:


(3) \begin{equation*} ECD=\sqrt{\sum_{i=1}^n\kern0.1em {\left({x}_i-1\right)}^2,} \end{equation*}

where $n$ is the number of codon preference and *x* is codon preference of different amino acids in the target species.

The codon adaptation index (CAI) [33] for each strain’s genome was calculated by the CAI Python package (version 1.0.3) [34]. The CAI values were obtained by comparing the RSCU values of each codon in a gene to those in a reference gene set and then taking the geometric mean, thereby indicating the adaptation level of a gene’s codon usage to the preferred codons of a highly expressed gene set. Additionally, we used the R package coRdon (version 1.22.0) [35] to calculate the E- and Fop-values [36, 37]. E-values were obtained by calculating the difference between the actual usage frequency and the theoretical uniformly distributed frequency of each synonymous codon family within a gene, followed by a weighted average. Fop-values were derived by determining the ratio of high-frequency codon usage within a gene to the total codon usage, thereby assessing the preference for optimal codons.

### Training of machine learning models

**Figure 2:**
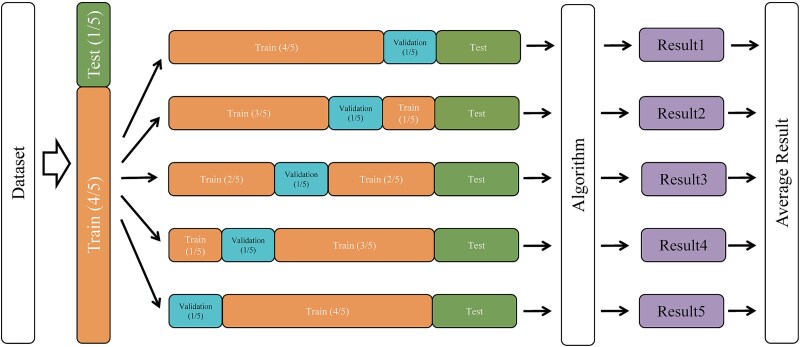
Five-fold cross-validation. Divide the dataset into training and test sets using a 4:1 ratio and then divide the training set into training and validation sets using a 4:1 ratio.

We divided the dataset into training and test sets using a 4:1 ratio. The training set was further split into training and validation subsets, also at a 4:1 ratio. We performed five-fold cross-validation on the training data, averaging the results from each fold to obtain the final model performance metrics (Fig. 2). This approach provided a robust evaluation by ensuring that each data subset was used for both training and validation across different iterations, reducing the risk of overfitting and providing a more reliable estimate of model accuracy.

### Evaluation and interpretability analysis of machine learning models

**Figure 3:**
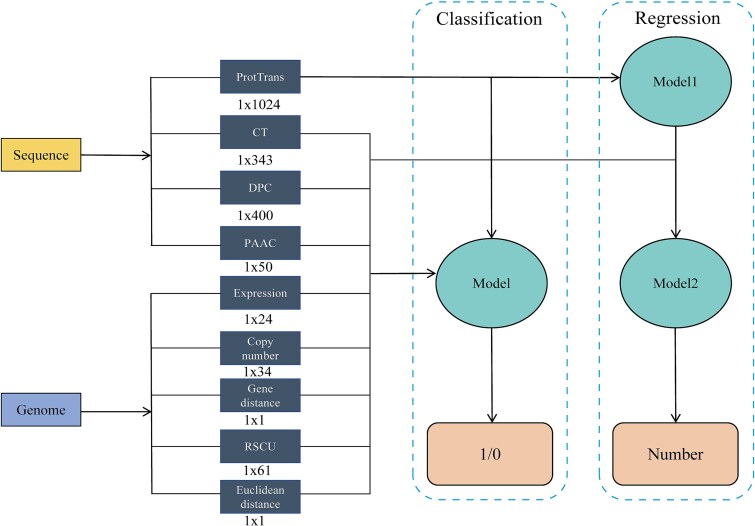
Flowchart of classification and regression models. Different models can be used depending on specific task (classification or regression).

For ML modeling of the extracted features, we employed several algorithms: random forest, support vector machine, XGBoost, Light Gradient Boosting Machine, Gradient Boosting Decision Trees, and multilayer perceptron (MLP). For regression tasks, we selected the top three performing models and further optimized them using a stacked model approach. In this stacked configuration, we first trained on the ProtT5-extracted features and used the initial predictions as inputs, combined with additional features, for a second-stage training. This two-step process enhanced model performance by leveraging ProtT5’s deep representation capabilities in combination with other feature sets, resulting in more accurate classification and regression outputs (Fig. 3). The specific feature extraction methods can be found in Supplementary Table S1.

To evaluate the performance of our classification models, we utilized metrics including area under receiver operating characteristic curve (AUC), precision, recall, and F1 score. For regression models, we assessed model quality using mean absolute error (MAE), mean squared error (MSE), root mean squared error (RMSE), and R^2^ values. To gain insights into the factors influencing nitrogenase activity predictions, we applied Shapley additive explanation (SHAP) analysis [38, 39]. SHAP provided interpretability by identifying key feature contributions, enhancing our understanding of the significant determinants within the predictive models.

## Results and discussion

### Data set overview

The distribution of the collected nitrogen-fixing bacterial species is shown in Table 1. The *Pseudomonadota* phylum has the highest representation, comprising 63.19% of the nitrogen-fixing bacteria, with *Alphaproteobacteria* being the predominant class within this group. Nitrogen-fixing bacteria in the *Alphaproteobacteria* class are notably widespread, thriving in diverse environments such as plant rhizospheres, deserts, and rice paddies [40, 41]. The *Bacillota* phylum accounts for 17.66%, while *Cyanobacteriota* represents 14.18% of the total. This distribution indicates a concentrated evolutionary pattern among nitrogen-fixing bacteria, suggesting a degree of evolutionary conservation in both the organisms and their nitrogenase [42].

**Table 1:** Distribution of nitrogen-fixing bacteria species

Phylum	Proportion(%)	Class	Number	Proportion(%)
*Actinomycetota*	2.74	*Actinomycetes*	11	2.74
*Bacillota*	17.66	*Bacilli*	70	17.41
		*Nitrospiria*	1	0.25
*Campylobacterota*	0.25	*Epsilonproteobacteria*	1	0.25
*Cyanobacteriota*	14.18	*Cyanophyceae*	57	14.18
*Nitrospirota*	0.25	*Negativicutes*	1	0.25
*Pseudomonadota*	63.19	*Alphaproteobacteria*	168	41.79
		*Betaproteobacteria*	36	12.44
		*Gammaproteobacteria*	50	8.96
*Thermodesulfobacteriota*	1.48	*Desulfuromonadia*	4	0.99
		*Desulfovibrionia*	2	0.49
*Verrucomicrobiota*	0.25	*Methylacidiphilae*	1	0.25

### Performance of classification models

**Figure 4:**
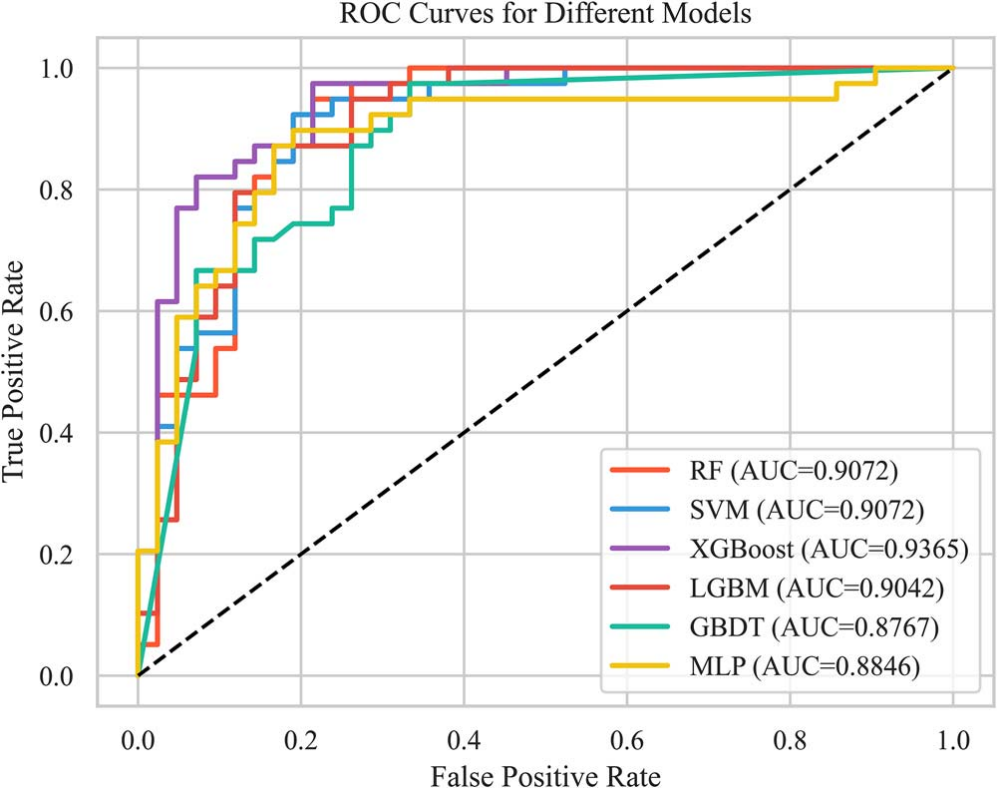
ROC curves for the six classification models on five-fold cross-validation.

Although both validation methods produced similarly average performances (Supplementary Tables S2 and S3) with the five-fold validation, the hold-out validation has high variance of performance (Figs S1 and S2) and the leave-one-out cross-validation involves with high time-consuming of calculations. The results of the five-fold cross-validation for various classification models are shown in Table 2, highlighting differences in performance across the training, validation, and test sets. These variations reflect the unique fitting and generalization characteristics of each algorithm. The XGBoost model performs exceptionally well on the training set, though it exhibits a slight decline on the validation and test sets. Notably, the fluctuations in precision point to a potential issue with overfitting. Despite this, XGBoost achieves the highest AUC score on the test set, indicating that it has the strongest generalization ability among all models when applied to new data. The receiver operating characteristic (ROC) curve is a valuable tool for assessing a model’s ability to distinguish between positive and negative samples across various threshold settings. However, in highly imbalanced datasets, the false positive rate can become very low, particularly when the number of negative samples is disproportionately large, which may reduce the curve’s sensitivity in representing performance. In our study, the dataset is relatively balanced, making the ROC curve a suitable choice for evaluation. As shown in Fig. 4, XGBoost achieved the highest AUC (0.9365) among all models, indicating superior discriminative ability and overall performance compared to other models in this experiment. The MLP model underperformed compared to tree-based models likely due to its need for larger datasets to optimize its numerous parameters and its lesser suitability for tabular data, whereas XGBoost excels with heterogeneous features typical in our dataset. Therefore, we recommend using this model when predicting nitrogenase activity type (strong or weak) in practical scenes.

**Table 2:** Five-fold cross-validation results for different classification models

Model	Dataset	AUC	Precision	Recall	F1
RF	Train	0.9986	0.9823	0.9838	0.983
	Validation	0.9215	0.8426	0.9222	0.8794
	Test	0.9072	0.8000	0.9231	0.8571
SVM	Train	0.9980	0.9823	0.9854	0.9838
	Validation	0.9268	0.7935	0.9409	0.8605
	Test	0.9072	0.8000	0.9231	0.8571
XGBoost	Train	0.9999	1	0.9919	0.9959
	Validation	0.9221	0.8540	0.8897	0.8696
	Test	**0.9365**	**0.8293**	**0.8718**	**0.8500**
LGBM	Train	0.9931	0.9433	0.9578	0.9503
	Validation	0.9096	0.8426	0.8570	0.8483
	Test	0.9042	0.7907	0.8718	0.8293
GBDT	Train	0.9999	0.9859	1	0.9928
	Validation	0.8810	0.7737	0.8370	0.8029
	Test	0.8767	0.7317	0.7692	0.7500
MLP	Train	0.9594	0.9388	0.8429	0.8813
	Validation	0.9091	0.8697	0.7901	0.8185
	Test	0.8846	0.8293	0.8718	0.8500

Bold is to emphasize that this set of data is the best, without any other meaning.

### Performance of regression models

The results of the five-fold cross-validation for various regression models are shown in Table 3. Each model displayed varying performance across the training, validation, and test sets, highlighting differences in fitting and generalization capabilities. The SVR model performed well on the training set, evidenced by a low MSE of 0.3463. However, its generalization declined considerably on the test set, with significantly higher RMSE and MAE values, indicating limitations in its applicability to new data. The XGBoost model showed an almost perfect fit on the training set, achieving a high R^2^ of 0.9896, but its R^2^ on the validation and test sets dropped to 0.4671 and 0.5133, respectively. Although this suggests severe overfitting, XGBoost still achieved a low RMSE of 0.5962 on the test set, indicating its ability to capture core data patterns to some extent. The MLP achieved an R^2^ of 0.7729 on the training set, reflecting good training fit, with moderate performance on the validation and test sets.

**Figure 5:**
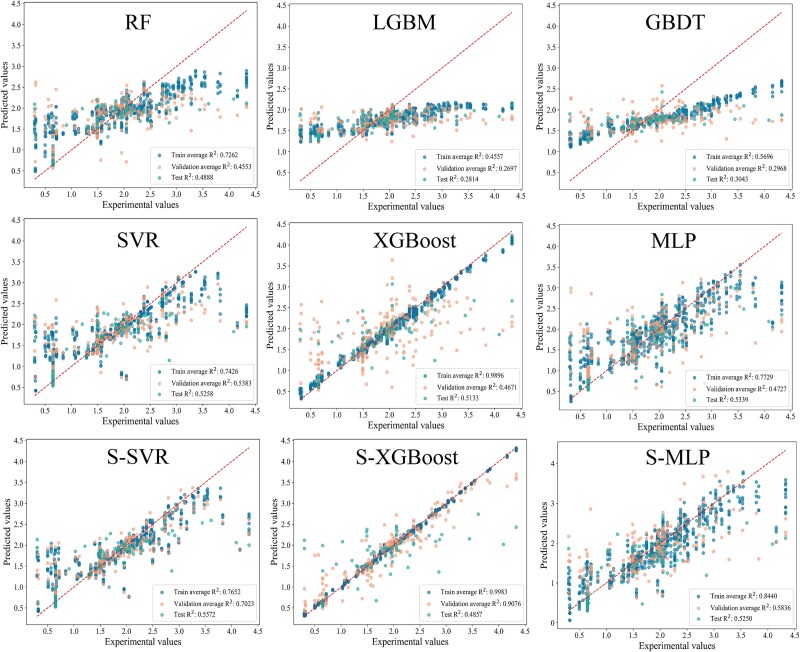
The predictive performance of the model is plotted. RF, random forest; GBDT, Gradient Boosting Decision Tree; LGBM, Light Gradient Boosting Machine; S-SVR, stacked SVR model; S-XGBoost, stacked XGBoost model; S-MLP, stacked MLP model.

Overall, SVR, XGBoost, and MLP demonstrated relatively better prediction accuracy and model generalization, performing well in the initial training round (Fig. 5).

### Optimization results for the regression models

For the three models (SVR, XGBoost, and MLP) that performed well in the initial round, we optimized them using stacked models. The first layer was trained with features extracted from ProtT5, and the results of the predictions from the first layer were used as inputs for the second layer, which was then trained with additional features. The training results of these stacked models are presented in Table 4. The S-SVR model achieved a high R^2^ of 0.7652 on the training set, demonstrating strong fitting performance. On the test set, S-SVR reached the highest R^2^ (0.5572) and the lowest MSE (0.3234), indicating it had the best performance. The S-XGBoost model performed excellently on both the training and validation sets, with an R^2^ of 0.9076 on the validation set and a significantly lower MSE (0.0527) than the other models, showcasing its ability to capture data patterns. However, its R^2^ on the test set dropped to 0.4857. This suggests that it suffered from serious overfitting. Some possible methods for reducing overfitting can be found in Supplementary Table S4. The performance of S-MLP was moderate, which was a little worse than S-SVR.

**Table 3:** Five-fold cross-validation results for different regression models

Model	Dataset	R^2^	RMSE	MSE	MAE
RF	Train	0.7262	0.4281	0.1834	0.2648
	Validation	0.4553	0.598	0.3638	0.3700
	Test	0.4888	0.6110	0.3734	0.3819
SVR	Train	0.7426	0.4150	0.1726	0.2208
	Validation	0.5383	0.5500	0.3065	0.3203
	Test	**0.5258**	**0.5885**	**0.3463**	**0.3343**
XGBoost	Train	0.9896	0.0831	0.0070	0.0586
	Validation	0.4671	0.5908	0.3598	0.3545
	Test	**0.5133**	**0.5962**	**0.3554**	**0.3614**
LGBM	Train	0.4557	0.6035	0.3645	0.4577
	Validation	0.2697	0.6932	0.4846	0.5209
	Test	0.2814	0.7244	0.5248	0.5538
GBDT	Train	0.5696	0.5367	0.2881	0.4294
	Validation	0.2968	0.6802	0.4675	0.5084
	Test	0.3043	0.7128	0.5081	0.5324
MLP	Train	0.7729	0.3880	0.1524	0.2579
	Validation	0.4727	0.5848	0.3472	0.3610
	Test	**0.5339**	**0.5834**	**0.3404**	**0.3580**

R^2^: Coefficient of determination.

**Table 4:** Five-fold cross-validation results for three stacked models

Model	Dataset	R^2^	RMSE	MSE	MAE
S-SVR	Train	0.7652	0.3962	0.1570	0.2121
	Validation	0.7023	0.4380	0.1939	0.2522
	Test	**0.5572**	**0.5687**	**0.3234**	**0.3351**
S-XGBoost	Train	0.9983	0.0325	0.0011	0.0218
	Validation	0.9076	0.2140	0.0527	0.1280
	Test	0.4857	0.6129	0.3756	0.3759
S-MLP	Train	0.8440	0.3224	0.1050	0.2340
	Validation	0.5836	0.5234	0.2795	0.3364
	Test	0.5250	0.5890	0.3469	0.3918

Bold is to emphasize that this set of data is the best, without any other meaning.

When comparing the stacked model performance to the original models, S-XGBoost showed a significant improvement on the validation set but a decrease on the test set, indicating increased overfitting. S-MLP did not show substantial improvement and instead showed a slight increase in error across all sets. On the other hand, S-SVR exhibited the most balanced performance. Although it did not excel in predicting extreme values, its overall performance across the training, validation, and test sets was consistent, making it the most suitable model for final predictions (Fig. 5).

### Interpretability analysis

**Figure 6:**
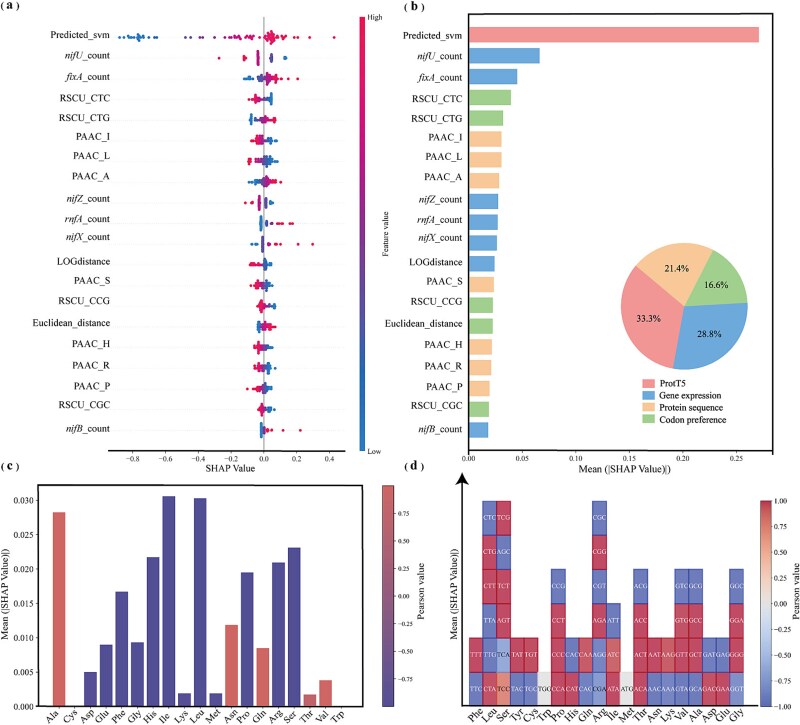
(a) Distribution of SHAP values for the 20 features with the greatest impact on the model prediction results; (b) characteristic importance of the 20 features with the greatest impact on the model prediction results; (c) importance of the 20 essential amino acid content features extracted by PAAC; (d) importance of the 20 essential amino acid codon preference features extracted by RSCU.

Through interpretable analysis of the S-SVR model, we identified key factors affecting nitrogenase activity and modeled a cluster of minimal nitrogen-fixing genes. The 20 most influential features affecting the predicted results of the model are displayed in Fig. 6a, and the complete SHAP analysis results are provided in Supplementary Table S5. The SHAP values indicate how each feature influences the model’s predictions. Features with SHAP values greater than 0 contribute positively to the target values (nitrogenase activity), while features with SHAP values less than 0 inhibit the target values. From the analysis, it is evident that the first layer predictions in the stacked model have the greatest impact on the results of the second layer (Fig. 6b), demonstrating that the feature values predicted by ProtT5 in the first layer are highly effective. This reflects the ability of the pretrained large model to accurately extract protein-related information.

#### Protein sequence characteristics

The sequence of a protein determines its structure, and the structure in turn determines its function, making the sequence information of nitrogenase fundamental to its activity [43]. Among the three methods for extracting protein sequences, the 20 basic amino acid content features extracted by PAAC have the greatest impact on model performance (Fig. S3). Figure 6c shows the mean of the absolute values of SHAP values for the 20 amino acid content profiles and their Pearson values in relation to the corresponding SHAP values. The results reveal that increasing the content of five amino acids promotes nitrogenase activity: Ala, Asn, Gln, Thr, and Val. The FeMo-cofactor is a nitrogenase complex containing the catalytic site [44], with Gln at position 191 of the FeMo-cofactor being the key site where nitrogenase catalyzes its reaction [45]. Our modeling results indicate that increasing the proportion of Gln promotes nitrogenase activity, confirming the model’s ability to capture the importance of Gln in nitrogen fixation catalysis. Additionally, amino acid substitution by Ala at position 70 of the FeMo-cofactor enhances nitrogen fixation efficiency [45]. Our SHAP analysis shows that increasing Ala content significantly boosts nitrogenase activity, aligning with literature reports [46, 47]. This suggests the model effectively captures the protein sequence factors influencing nitrogenase activity. The SHAP analysis also identifies three other amino acids whose increased content promotes nitrogenase activity, offering potential targets for genetic engineering in nitrogen-fixing bacteria. When the content of polar and neutral amino acids (Asn, Gln) is increased, nitrogenase activity is optimized. In contrast, higher levels of nonpolar amino acids (Ile, Pro, Phe) result in decreased nitrogenase efficiency. This could be related to the fact that nitrogen fixation involves numerous electron transfers, and the side chains of polar amino acids can stabilize these processes through hydrogen bonding and electrostatic interactions. These interactions help form stable bonds with cofactors (iron–sulfur clusters, flavins) or molecules involved in electron transfer, thereby enhancing electron transfer efficiency [48]. Furthermore, nitrogen fixation enzymes have a preference for Asn and Gln, suggesting that the amide group plays an essential role in nitrogen fixation. This may be linked to the process where ammonia, after being immobilized and produced, binds to other compounds through amidation reactions [49].

#### Gene expression and gene distance

The nitrogen fixation efficiency of nitrogenase is strongly influenced not only by their sequence characteristics but also by the enzyme quantity. To enhance model accuracy and minimize missing values, we employed the three methods (E, Fop, CAI) to predict gene expression only for proteins that had relevant gene annotations in the genomes of species representing over 95% of the dataset (*lnA*, *nifB*, *nifD*, *nifH*, *nifK*, *nifE*, *nifN*, *nifX*). From Fig S4, it is evident that Average_*nifK*_E and Average_*nifD*_E significantly influence the model results, with both showing a positive correlation with the SHAP values. The *nifD* and *nifK* genes encode a molybdenum–iron protein that contains the key active site, the FeMo-cofactor, which serves as the central catalytic site for nitrogen reduction in nitrogenase [44]. The model further confirms that the expression levels of *nifD* and *nifK* have a substantial impact on the nitrogen fixation efficiency of nitrogen-fixing bacteria, suggesting that the nitrogen fixation efficiency may indeed be linked to the varying expression levels of these two genes in different nitrogen-fixing bacteria.

The physical location of a gene in the genome, along with its distance from other genes or regulatory regions, significantly influences gene expression. The relative positioning of genes within a cluster affects their proximity to regulatory regions, thereby influencing their expression levels [50]. In our analysis, we included the gene distances of *nifD*, *nifK*, and *nifH* within the genome as part of the feature set. From Fig. S4, it is evident that this feature ranks highly and has a substantial effect on the nitrogen fixation efficiency of nitrogenase.

#### Codon preference

In this study, we incorporated codon preferences in nitrogen-fixing bacteria as part of the characterization process and calculated the ECD relative to the genome without considering codon preferences [51]. As shown in Fig. S5, the ECD significantly influences nitrogen fixation efficiency. Notably, as the ECD increases, there is a more pronounced improvement in nitrogen fixation efficiency. A higher ECD indicates stronger codon preference in the bacterial genome, which is typically associated with faster growth rates. This suggests that the growth rate of nitrogen-fixing bacteria has a substantial impact on nitrogen fixation efficiency and faster-growing bacteria exhibit enhanced nitrogen fixation activity. The 61 codons that encode the 20 standard amino acids (excluding the three stop codons) often exhibit redundancy, where multiple codons encode the same amino acid. This phenomenon, known as codon concatenation [52], plays a key role in the efficiency of nitrogen fixation. Figure 6d summarizes the relationship between codon preferences and SHAP values for the 20 standard amino acids, showing that nitrogen-fixing bacteria generally favor most standard amino acids, with the exception of Trp and Met. Additionally, key residues involved in catalysis within the nitrogen fixation enzyme complex include Asp and Lys in *nifH* [53]. The dominant codons GAC and AAG can be utilized to synergistically improve nitrogen fixation efficiency.

#### Construction of a cluster of minimal *nif* genes

**Figure 7:**
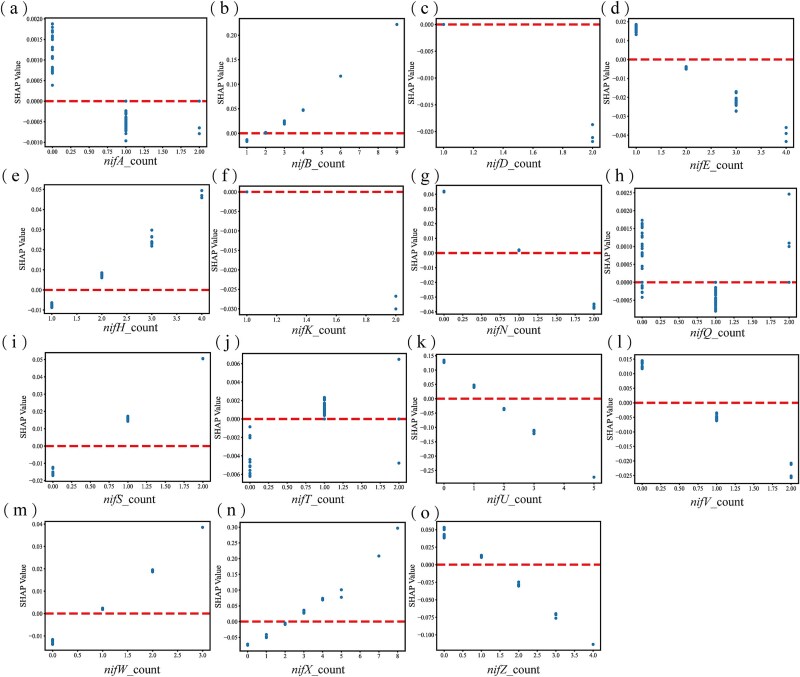
The relationship between copy number of different *nif* genes and their SHAP values (a–o).

Nitrogen-fixing gene clusters can be transferred and expressed in non-nitrogen-fixing organisms, enabling these organisms to acquire nitrogen-fixing capabilities [54]. In recent years, numerous *nif* genes from nitrogen-fixing strains have been successfully transferred to *Escherichia coli*, resulting in the expression of nitrogen-fixing activity [55]. In this study, we further analyzed the copy number characteristics of *nif* genes and their SHAP values, aiming to identify the minimal nitrogen-fixing *nif* gene cluster. All *nif* genes present in more than half of the nitrogen-fixing bacteria were selected, and the relationship between their copy number and SHAP value is shown in Fig. 7.

The final minimal *nif* gene cluster contains one copy number of *nifD*, *nifK*, *nifE*, *nifS*, *nifT*, *nifW*, and two copy numbers of *nifH*, *nifB*, and three copy numbers of *nifX* (Fig. S6). Five conserved genes, *nifH*, *nifD*, *nifK*, *nifE*, and *nifB*, form the catalytic core required for the *in vitro* assembly of FeMo-co [56]. The minimal nitrogen-fixing cluster identified in our study offers new insights and guidance for transferring nitrogen-fixing capacity.

### Brief discussion and perspective

AI is developing rapidly, but it has not been widely applied in the field of nitrogen fixation engineering. In our study, we first build Carmna using ProtTrans, an advanced tool based on NLP for proteins, which greatly improved the accuracy of nitrogenase feature extraction. Although generalized large models have good performance, current research lacks excellent models for specific areas. Our work integrates nitrogenase data from the existing literature, which is not only used to build our model but also facilitates future research by allowing researchers to easily access nitrogenase activity data from the repository for further study. In addition, SHAP analysis allowed us to obtain information on the amino acid composition [46, 47] that affects nitrogenase activity and supplied the computational simulation of minimal nitrogen-fixing gene clusters, which previously often required extensive experimentation. For the exploration of minimal nitrogen fixation gene clusters, Harris demonstrated nitrogen-fixing activity in *E. coli* by introducing the *nifHDKTYENXUSVWZMF* cluster [57], while Wang proposed a minimal nitrogen fixation cluster consisting of *nifB*, *nifH*, *nifD*, *nifK*, *nifE*, *nifN*, *nifX*, *hesA*, and *nifV* from *Paenibacillus sp.* WLY78 [58]. Compared to Harris’ study, we reduced redundant nitrogen fixation genes to identify the minimal nitrogen fixation gene cluster. Relative to Wang’s work, we also accounted for the types and copy numbers of *nif* genes in the minimal cluster, optimizing the design to ensure better functional interactions among nitrogenase proteins. Our work will facilitate high-throughput screening for efficient nitrogenase and provide guidance for directed evolution of efficient nitrogenase.

However, our study has certain limitations. The dataset on nitrogenase activity is currently insufficient to cover a broad range of conditions, and the model’s predictive accuracy for extreme values of nitrogen fixation activity remains limited. Despite these constraints, our work contributes novel insights to the field of bio-fertilizer design. In future research, we aim to expand the dataset on nitrogenase activity and refine the prediction algorithm to enhance its performance. We believe that advancements in technology will ultimately enable Carmna to provide more value to the research and applications in bio-fertilizers.

## Conclusion

In this study, we developed Carmna that contain classification and regression models to predict nitrogenase activity by applying six ML methods. We utilized the pretrained ProtT5 model for extracting sequence features and incorporated additional variables such as gene expression and codon preference. We proposed XGBoost model for classification tasks and the stacked SVR model for regression tasks. Interpretive analysis of the top-performing regression model revealed five basic amino acid compositions that positively impact nitrogenase activity, clarified how multiple expressions of the *nifD* and *nifK* genes contribute to higher nitrogen fixation rates, and summarized the distinct effects of 61 codon preferences across 20 standard amino acids on nitrogenase activity. We also identified the smallest functional *nif* gene cluster required for nitrogen fixation. These findings provide valuable insights into optimizing the genetic engineering of nitrogen-fixing bacteria, supporting selection and construction of high-efficiency nitrogen-fixing bacterial strains and fostering advancements in development and application of bio-fertilizers.

**Key Points**

- This is the first time to build classification and regression models for predicting nitrogenase activity (Carmna) values using six machine learning algorithms.
- XGBoost performed best in classification models, while the stacked support vector regression model performed best in regression models.
- Carmna identified five standard amino acids that enhance nitrogenase activity, analyzed the effect of 61 codon preferences on nitrogenase activity and determined the minimal set of *nif* genes necessary for nitrogen fixation.

## Supplementary Material

Supplement_material_bbaf197

Table_S5_Complete_SHAP_analysis_bbaf197

## Data Availability

The information of dataset and models can be found on GitHub (https://github.com/AnqiangYe/nitrogenase).
