# Supplementary material for: Carmna: classification and regression models for nitrogenase activity based on a pretrained large protein language model
Source: Brief Bioinform. 2025 Apr 24;26(2):bbaf197. doi: 10.1093/bib/bbaf197 (PMC12021265; doi:10.1093/bib/bbaf197)
Supplement: Supplement_material_bbaf197 [file supplement_material_bbaf197.docx]

**Carmna: classification and regression models for nitrogenase activity on a pre-trained large protein language model**

**
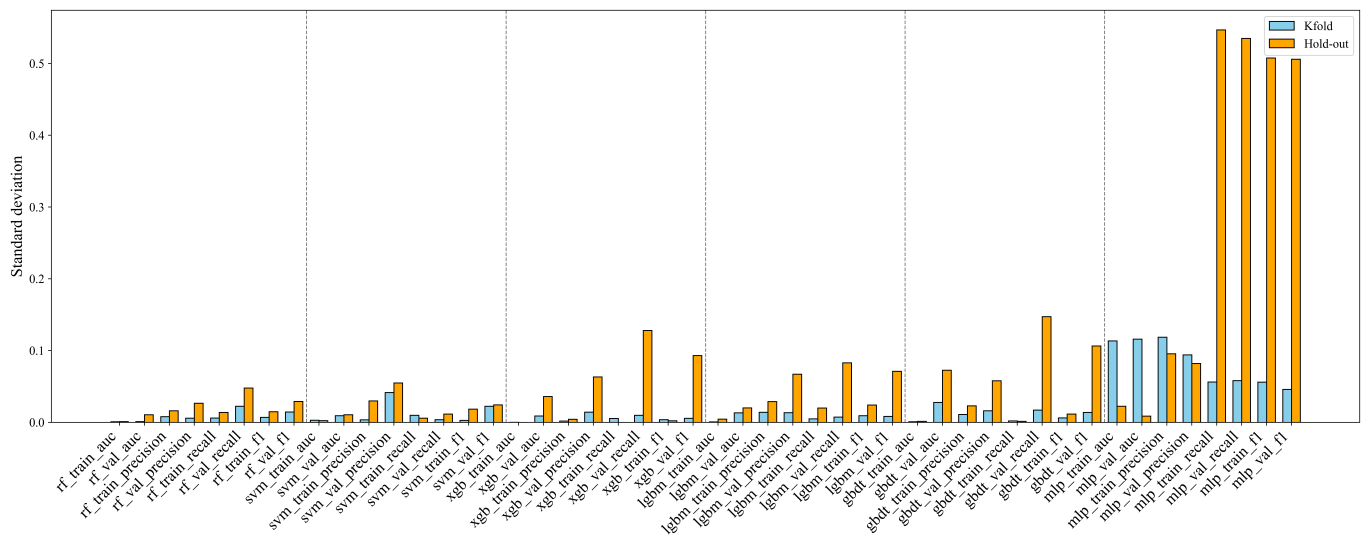
**

**Fig. S1.** **Standard deviation using hold-out and k-fold validation in classification models**.

**
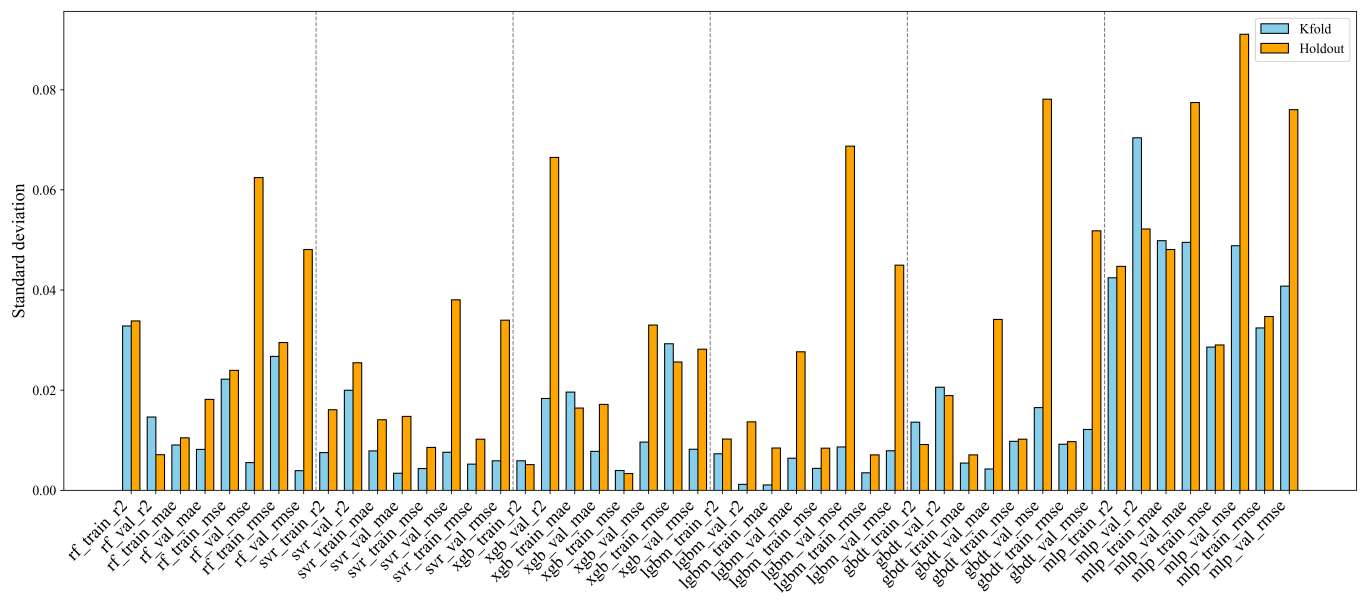
**

**Fig. S2.** **Standard deviation using hold-out and k-fold validation in regression models**.


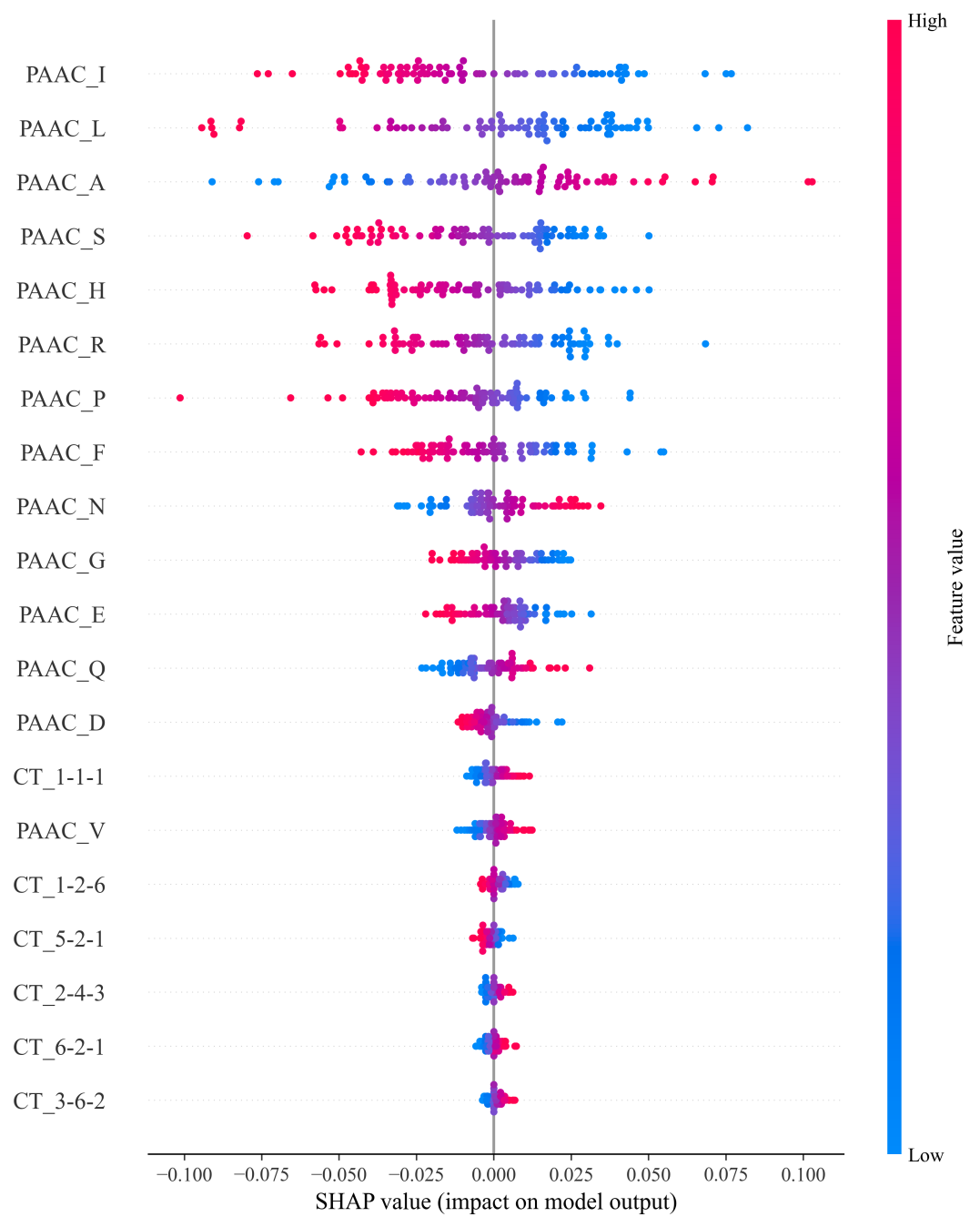


**Fig. S3.** **Distribution of SHAP values for protein sequence characteristics (top 20).**


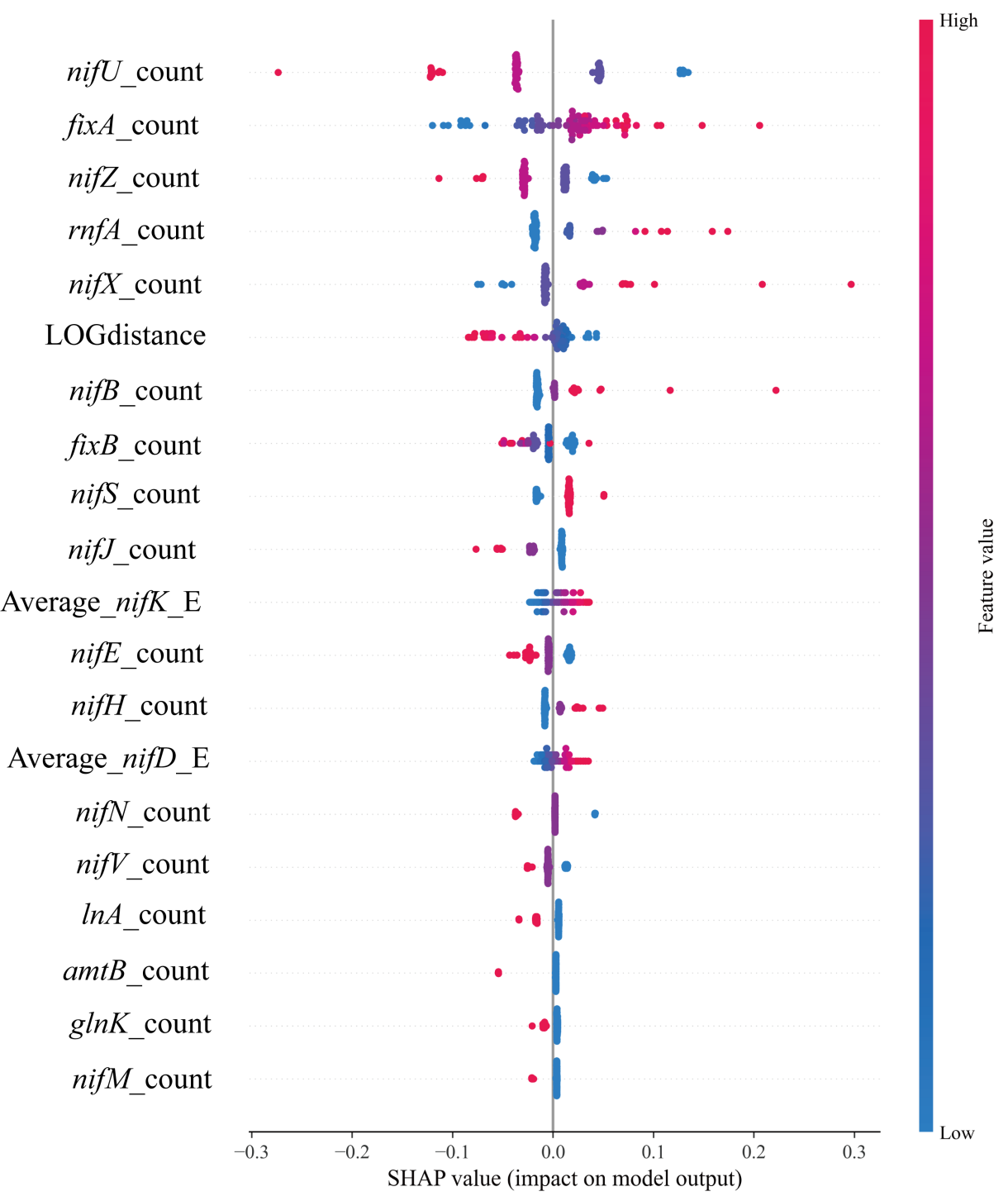


**Fig. S4.** **Distribution of SHAP values for gene expression and gene distance (top 20).**


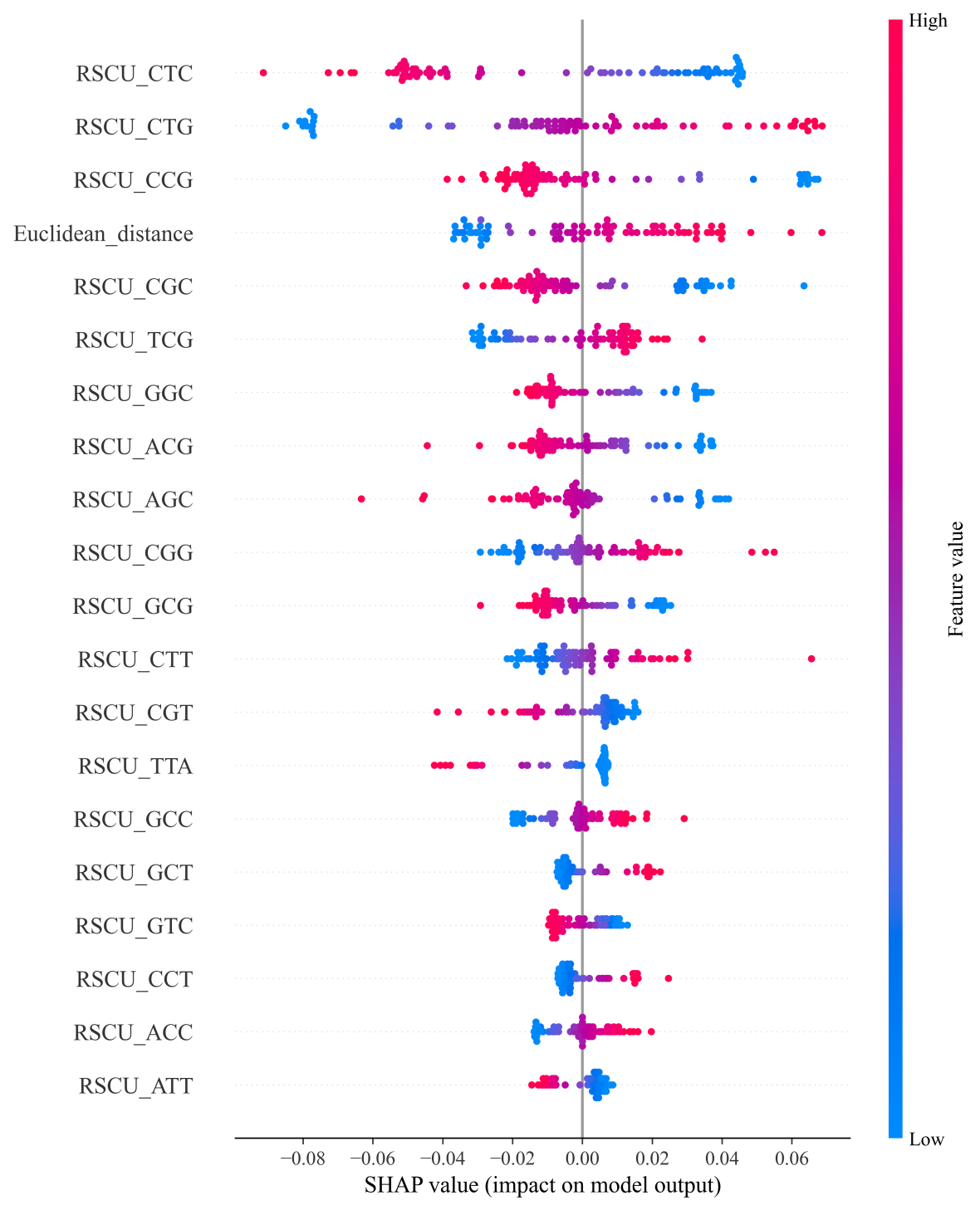


**Fig. S5.** **Distribution of SHAP values for codon preference (top 20).**


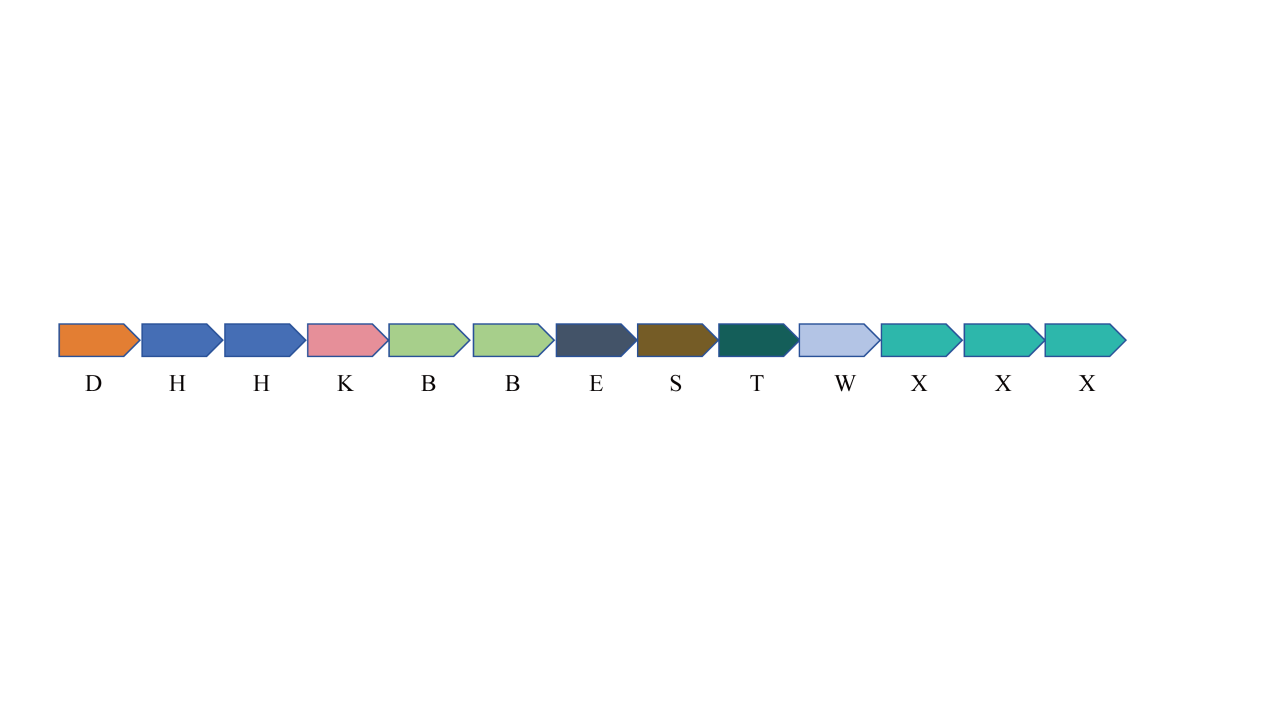


**Fig. S6.** **The minimal nitrogen fixation *nif* gene cluster.**

**Table S1.** Summary of feature extraction methods

| Feature | Software | Dimension | Detail step |
| --- | --- | --- | --- |
| ProtTrans | ProtT5 | 1x1024 | Extracting features of nitrogenase sequences (*nifD*, *nifH*, *nifK*) using module “ Embedding Section” in ProtT5. Take the average of the three nitrogenase features obtained to obtain the final feature value. |
| CT |  | 1x343 | Extracting features of nitrogenase sequences (*nifD*, *nifH*, *nifK*) using the methods described in the literature^1*^. Take the average of the three nitrogenase features obtained to obtain the final feature value. |
| DPC |  | 1x400 | Extracting features of nitrogenase sequences (*nifD*, *nifH*, *nifK*) using the methods described in the literature^2*^. Take the average of the three nitrogenase features obtained to obtain the final feature value. |
| PAAC |  | 1x50 | Extracting features of nitrogenase sequences (*nifD*, *nifH*, *nifK*) using the methods described in the literature^3*^. Take the average of the three nitrogenase features obtained to obtain the final feature value. |
| Expression | CAI (version 1.0.3);  coRdon (version 1.22.0) | 1x24 | Calculate the CAI, E, Fop values of 8 nitrogen fixing genes that are present in most nitrogen fixing microorganisms^4*^. CAI value was calculated by CAI. E and Fop values were calculated by coRdon. |
| Copy number |  | 1x34 | Calculate the gene copy numbers of 34 nitrogen fixation process related genes^5*^. |
| Gene distance |  | 1x1 | Calculate the distance between *nifD*, *nifH*, *nifK* genes in the genome. |
| RSCU | CAI (version 1.0.3) | 1x61 | Using CAI software to calculate RSCU. |
| Euclidean distance |  | 1x1 | Calculated the Euclidean distance of the target species relative to the standard species without codon preference (all codon preference values are 1). |

^1*^Shen, J., Zhang, J., Luo, X., Zhu, W., Yu, K., Chen, K., Li, Y. and Jiang, H., 2007. Predicting protein–protein interactions based only on sequences information. *Proceedings of the National Academy of Sciences*, *104*(11), pp.4337-4341.

^2*^Bhasin, M. and Raghava, G.P., 2004. Classification of nuclear receptors based on amino acid composition and dipeptide composition. *Journal of Biological Chemistry*, *279*(22), pp.23262-23266.

^3*^Chou, K.C., 2001. Prediction of protein cellular attributes using pseudo‐amino acid composition. *Proteins: Structure, Function, and Bioinformatics*, *43*(3), pp.246-255.

^4*^The 8 nitrogen fixing genes that are present in most nitrogen fixing microorganisms: *lnA*, *nifB*, *nifD*, *nifE*, *nifH*, *nifK*, *nifN*, *nifX*.

^5*^The 34 nitrogen fixation process related genes: *nifD*, *nifH*, *nifK*, *amtB*, *fixA*, *fixB*, *fixC*, *fixX*, *glnK*, *lnA*, *nifA*, *nifB*, *nifE*, *nifF*, *nifJ*, *nifL*, *nifM*, *nifN*, *nifP*, *nifQ*, *nifS*, *nifT*, *nifU*, *nifV*, *nifW*, *nifX*, *nifY*, *nifZ*, *rnfA*, *rnfB*, *rnfC*, *rnfD*, *rnfE*, *rnfG*.

**Table S2.** **Results of** **Leave-One-Out Cross-Validation (LOOCV) for classification models**

| Model | Dataset | AUC | Precision | Recall | F1 |
| --- | --- | --- | --- | --- | --- |
| RF | Train | 0.9974 | 0.9678 | 0.9742 | 0.9710 |
|  | Validation | 0.9264 | 0.8537 | 0.9091 | 0.8805 |
|  | Test | 0.9096 | 0.7872 | 0.9487 | 0.8605 |
| SVM | Train | 0.9972 | 0.9807 | 0.9870 | 0.9838 |
|  | Validation | 0.9274 | 0.8 | 0.9610 | 0.8732 |
|  | Test | 0.9231 | 0.7451 | 0.9744 | 0.8444 |
| XGBoost | Train | 0.9996 | 0.9857 | 0.9815 | 0.9836 |
|  | Validation | 0.9136 | 0.8608 | 0.8831 | 0.8718 |
|  | Test | 0.9286 | 0.8095 | 0.8718 | 0.8395 |
| LGBM | Train | 0.9928 | 0.9485 | 0.9513 | 0.9499 |
|  | Validation | 0.9105 | 0.8323 | 0.8701 | 0.8508 |
|  | Test | 0.9103 | 0.8 | 0.8205 | 0.8101 |
| GBDT | Train | 0.9988 | 0.9585 | 0.9810 | 0.9696 |
|  | Validation | 0.9047 | 0.8571 | 0.8961 | 0.8762 |
|  | Test | 0.8208 | 0.8182 | 0.6923 | 0.7500 |
| MLP | Train | 0.9433 | 0.8838 | 0.8756 | 0.8797 |
|  | Validation | 0.8870 | 0.8425 | 0.7987 | 0.8200 |
|  | Test | 0.8944 | 0.7551 | 0.9487 | 0.8409 |

**Table S3.** **Results of** **Leave-One-Out Cross-Validation (LOOCV) for regression models**

| Model | Dataset | R^2^ | RMSE | MSE | MAE |
| --- | --- | --- | --- | --- | --- |
| RF | Train | 0.6941 | 0.4528 | 0.2050 | 0.2730 |
|  | Validation | 0.4734 | 0.5940 | 0.3528 | 0.3589 |
|  | Test | 0.4824 | 0.6148 | 0.3780 | 0.3708 |
| SVR | Train | 0.7375 | 0.4194 | 0.1759 | 0.2211 |
|  | Validation | 0.5626 | 0.5414 | 0.2931 | 0.3133 |
|  | Test | 0.5798 | 0.5540 | 0.3069 | 0.3310 |
| XGBoost | Train | 0.9824 | 0.1085 | 0.0118 | 0.0749 |
|  | Validation | 0.5022 | 0.5775 | 0.3335 | 0.3583 |
|  | Test | 0.5366 | 0.5817 | 0.3384 | 0.3540 |
| LGBM | Train | 0.4657 | 0.5983 | 0.3580 | 0.4554 |
|  | Validation | 0.3022 | 0.6838 | 0.4676 | 0.5117 |
|  | Test | 0.3231 | 0.7031 | 0.4944 | 0.5404 |
| GBDT | Train | 0.5493 | 0.5495 | 0.3020 | 0.4364 |
|  | Validation | 0.3259 | 0.6721 | 0.4517 | 0.5016 |
|  | Test | 0.3339 | 0.6975 | 0.4864 | 0.5244 |
| MLP | Train | 0.7185 | 0.4343 | 0.1886 | 0.2872 |
|  | Validation | 0.5129 | 0.5713 | 0.3264 | 0.3734 |
|  | Test | 0.5543 | 0.5705 | 0.3255 | 0.3801 |

**Table S4.** Some possible methods for reducing overfitting

| Method | Description |
| --- | --- |
| Proper regularisation strength | Systematically search for the regularization parameters of the model (reg_alpha, reg_lambda) to find the appropriate regularization strength that minimizes overfitting. |
| Model complexity reduction | Explore reducing the maximum depth of the trees (max_depth) and the number of trees (n_estimators) to simplify the model and improve its generalisation ability. |
| Early stopping | Implement early stopping during training to prevent the model from learning noise in the training data. This can be achieved by monitoring the validation performance and stopping training when performance plateaus. |
| Hyperparameter tuning | Systematically optimise hyperparameters such as learning_rate, max_depth, reg_alpha and reg_lambda to find the best combination that balances bias and variance. |
| Feature selection | Perform feature selection to remove irrelevant or redundant features, which can help reduce overfitting and improve model interpretability. |
| Dataset updating | Use artificial intelligence tools to collect nitrogenase activity data in the literature more comprehensively, and more data may be beneficial in mitigating the overfitting phenomenon. |
